# More sustainable choices in the workplace: a systematic review of nudge theory applications

**DOI:** 10.3389/fpsyg.2025.1556796

**Published:** 2025-08-20

**Authors:** Giulia De Paolis, Lorenza Tiberio, Federica Caffaro

**Affiliations:** Department of Education, Roma Tre University, Rome, Italy

**Keywords:** nudge theory, sustainable behavior, workplace, sustainable organizations, scoping review

## Abstract

Nudge theory proposes subtle changes to the choice environment to influence behavior without restricting autonomy. This scoping review investigates the application of nudging strategies within workplace settings to promote pro-environmental behaviors among employees. Sixteen peer-reviewed empirical studies were selected using PRISMA guidelines from four major databases. The analysis examines the characteristics of the selected studies, theoretical definitions of nudging, intervention types, and observed effectiveness. Findings show a notable increase in publications from 2020 onward, reflecting growing academic and institutional interest in sustainable organizational practices. Most studies are based in high-income Western countries, particularly within the tertiary sector. A clear heterogeneity emerged in how nudging is defined, with only half of the studies explicitly referencing Thaler and Sunstein’s classical framework. Others relied on broader psychological theories, such as social norms or cognitive biases, often without operational clarity. The nudges identified were classified into five categories: informational, reminders and notifications, financial incentives, non-financial incentives, and positioning and default options. Informational nudges and reminders were most commonly applied due to their low intrusiveness and implementation costs, but positioning and incentive-based strategies showed promising results in certain organizational contexts. Effectiveness was highest when nudges were paired with tangible incentives, required minimal effort, and were socially supported by leadership or peers. Conversely, interventions targeting high-cost behaviors or lacking enabling infrastructure were less successful. A lack of long-term follow-up assessments also limits conclusions about the durability of effects. This review highlights the need for consistent operational definitions, context-sensitive design, and longitudinal research. It emphasizes the importance of integrating behavioral insights into organizational structures and processes to foster environmentally responsible behaviors at work and beyond.

## 1 Introduction

Corporate sustainability is grounded in three interdependent pillars - environmental, social, and economic- which organizations operationalize through Corporate Social Responsibility (CSR) practices. These practices have traditionally been framed by Carroll’s pyramid of economic, legal, ethical, and philanthropic responsibilities, and more recently by Environmental, Social, and Governance (ESG) frameworks, which offer standardized metrics for assessing social and environmental impacts and risks (Carroll, 2016). This commitment is further reinforced by the European regulatory context, particularly the Corporate Sustainability Reporting Directive (CSRD, 2022/2464/EU), which introduces more stringent disclosure requirements as of 2024, and Regulation (EU) 2020/852, the so-called “Taxonomy,” which establishes unified criteria for identifying economically sustainable activities (Baks, 2024; Lámfalusi et al., 2024; Operato et al., 2025). However, the authenticity and credibility of any CSR–ESG agenda cannot rely solely on outward-facing initiatives. If sustainability values are not internalized within the organization, such efforts risk becoming mere reputation management. Only through a genuine transformation of its operating models, which fully embody ESG and CSR principles, can a company truly achieve credible social responsibility towards the community (Lu et al., 2020). For this reason, it is essential to actively engage employees and foster bottom-up behaviors aligned with both regulatory and strategic sustainability objectives. In the workplace context, environmentally sustainable behaviors may include daily practices such as waste sorting, energy conservation, the use of sustainable transportation, and participation in organizational initiatives aimed at reducing environmental impact, such as eco-design or carbon footprint programs.

Behavioral approaches that encourage sustainable choices using non-coercive and low-cost methods are receiving increasing attention to facilitate this internal transformation. A key framework in this area is the Nudge Theory, developed by Thaler and Sunstein (2008) which argues that even small changes to people’s decision-making environment, or “choice architecture,” can have a significant impact, without limiting their freedom of choice.

Although nudge-based interventions have been widely studied, a comprehensive synthesis of research examining their use in workplace environments to advance environmental sustainability is still missing. Establishing a comprehensive overview of existing literature is essential, not only to map current knowledge and identify key trends, but also to inform and guide future empirical studies. This manuscript aims to fill this gap by providing a scoping review of empirical research that applies nudge theory to incentivize more sustainable choices in organizational settings.

### 1.1 The nudge theory

The use of sanctions or prohibitions, which are often applied in practice, can be counterproductive in promoting pro-environmental behaviors and encouraging their internalization as habits or conscious choices, since such behaviors tend to decrease once the coercive measures have been eliminated (Moratti, 2020). In contrast, studies on Nudge Theory have been implemented to improve pro-environmental behaviors, by using subtle suggestions and designing choice architectures.

According to Thaler and Sunstein (2008, p. 6), a nudge is *“any aspect of the choice architecture that alters people’s behavior in a predictable way without forbidding any options or significantly changing their economic incentives. To count as a mere nudge, the intervention must be easy and cheap to avoid. Nudges are not mandates.”* Nudge theory is based on the notion that human rationality is limited. According to Kahneman (2011), despite our perception of being rational and deliberate thinkers, much of our daily decision-making relies on quick, intuitive judgments rather than careful analysis. This automatic mode of thinking, characterized by speed and efficiency, draws on past experiences and mental shortcuts, allowing us to navigate familiar situations with ease. In this dual-process framework, Nudge Theory aims to influence automatic behavior through implicit cues or environmental changes (Congiu and Moscati, 2020; Li and Chapman, 2013).

Nudging has been used to promote the adoption of pro-environmental behaviors in different contexts, without limiting behavioral alternatives but by making pro-environmental behaviors easier, more appealing, and more intuitive (Mont et al., 2014; Thaler and Sunstein, 2008; Kahneman, 2011). For instance, eco-labels and strategic product placement have been shown to increase the selection of low-impact food items (Wongprawmas et al., 2023; Lehner et al., 2016), while real-time feedback mechanisms - such as energy-consumption monitors and recycling reminders - facilitate the reinforcement of pro-environmental habits over time (Wong-Parodi et al., 2020; Santos, 2022). Social-comparison messages that convey peer conformity to eco-friendly norms exert a substantial influence on individual behavior, particularly in contexts where social approval is paramount (Amiri et al., 2024). Collective initiatives, exemplified by the “Sustainability Walk” (Westin et al., 2024) further demonstrate that the integration of nudges with participatory activities can amplify engagement in group-level environmental actions.

However, the term “nudge” is often used as an umbrella concept, encompassing a broad array of behavioral-economics techniques (Kay et al., 2024), which can lead to conceptual and methodological ambiguity. Not all interventions labeled as nudges in several studies fully meet the criteria defined by Thaler and Sunstein, leading to confusion between nudges and more explicit behavioral prompts (Grilli and Curtis, 2021; Ledderer et al., 2020). Nonetheless, fundamental differences exist between these approaches, primarily related to how the information is presented and the context in which the intervention is implemented (Rondina et al., 2022). Nudges operate at an implicit level, influencing behavior through subtle modifications to the choice environment and relying on automatic processing (e.g., defaults, visual salience, cognitive laziness) (Tagliabue et al., 2019; Thaler and Sunstein, 2008). In contrast, prompts aim to elicit conscious, intentional responses, requiring active cognitive effort and motivation (Mehr et al., 2020; Van Kleef et al., 2015). The effective design of nudges requires not only a deep understanding of cognitive mechanisms (Sunstein, 2014; Li and Chapman, 2013; Chandrashekar and Fillon, 2024), but also a careful assessment of the specific environmental and social context in which the intervention is implemented (Loan and Balanay, 2022; Gonzalez and Hinestroza, 2024). With regard to this latter point, the organizational context has been underinvestigated in literature, despite the central role that workplace behaviors play in achieving organizational sustainability.

Actually, there is some empirical evidence that nudges may support sustainable innovation at work (Brones et al., 2014), but most nudging interventions have targeted consumers rather than employees (Sharma et al., 2023; Tikotsky et al., 2020). Falcone and Fiorentino (2024) and most studies emphasized short-term outcomes, often overlooking whether nudges produce behavioral changes over time (Amiri et al., 2024; Forberger et al., 2021). This highlights the need for longitudinal and context-sensitive studies to assess the durability and generalizability of nudging interventions (Amiri et al., 2024).

### 1.2 Aims of the present study and research questions

Considering nudging strategies’ potential to support behavioral change, it is essential to develop a deeper understanding of how to effectively implement them also in the workplace. Based on these considerations, this study conducts a scoping literature review of nudge interventions targeted at organizational settings to encourage pro-environmental behaviors among employees.

To reach this aim, the study addressed the following research questions:

RQ1. What are the main characteristics of the available studies on nudging to promote workers’ pro-environmental behaviors at work (e.g., publication year, country, sample size, and productive sector)?
  Answering this question may help trace the evolution of research in this area, identify underexplored contexts, and suggest directions for future empirical studies.
RQ2. How do available studies define and categorize their interventions as nudges?
  By examining the theoretical definitions authors invoke when labeling interventions as “nudges,” we will help clarify conceptual inconsistencies and establish a clear taxonomy for future research and practice.
RQ3. What types of nudges are used in workplace settings?
  By answering this question, we will map the most prevalent choice-architecture tools which can be used to promote pro-environmental behaviors among workers.
RQ4. Are these nudges effective in promoting sustainable behaviors among workers?
  By considering the effectiveness of nudging interventions, we can identify their limitations and successes, guiding practitioners toward evidence-based strategies that maximize environmental benefits.

## 2 Materials and methods

### 2.1 Search strategy

The review process was carried out following the PRISMA (Preferred Reporting Items for Systematic Reviews and Meta-Analyses) guidelines (Moher et al., 2015; Shamseer et al., 2015). The literature search was conducted in May 2025. Articles were retrieved from four widely recognized databases: Scopus, PubMed, Web of Science, and PsycInfo. The search string ‘nudg* AND (sustainab* OR environment* OR green) AND (behavio* OR conduct* OR act* OR practice*) AND (work* OR organization*)’ was used to identify relevant articles from 2008 to 2025. This timeframe was chosen to align with the initial definition of the nudge concept, introduced with the publication of the foundational work by Thaler and Sunstein (2008), which laid the groundwork for this concept. Keywords were selected from our theoretical framework to align with the research objectives and included domain-specific terms (e.g., “nudg*” “sustainab*, “organization*”), including synonymous words, such as behavio*, conduct*, act* and practice*, in order to ensure semantic breadth and avoid missing relevant studies due to variations in terminology. These terms were then manually tailored to each database’s indexing fields (titles, abstracts, keywords or all fields) and syntax to maximize coverage and precision.

We defined the inclusion criteria (ICs) using the PICOS framework, with additional time-frame and language restrictions. Publications were included if they met the following inclusion criteria (ICs):

IC1. Population: Articles that involve adult workers (≥ 18 years) employed in public or private organizational settings.
IC2. Intervention: Articles that used nudging techniques to promote pro-environmental behaviors in the workplace.
IC3. Outcomes: Articles that measure changes in workplace pro-environmental behaviors (e.g., reduced resource consumption, increased recycling).
IC4. Study Design: Articles that report empirical studies.
IC5. Time Frame: Articles that were published between 2008 and 2025.
IC6. Language: Articles that are available in full-text in English.

Papers that fulfilled at least one of the following exclusion criteria (ECs) were excluded:

EC1. Conference papers, book chapters or reviews.
EC2. Short papers (1–4 pages, e.g., extended abstract or research in progress).
EC3. Articles that present prototypes or frameworks.
EC4. Articles without empirical data.
EC5. Articles that mention nudging but nudges are not part of the research being conducted.

We applied a four-stage screening procedure: (1) removal of duplicates and exclusion of non-English records; (2) title, keyword and abstract screening to identify potentially relevant studies; (3) full-text review of abstracts and articles against our inclusion criteria; and (4) a final consensus meeting to confirm the studies selected. The screening was performed independently by the three authors, and any disagreements concerning the inclusion of articles in this review were addressed and solved when reaching consensus.

**Figure 1:**
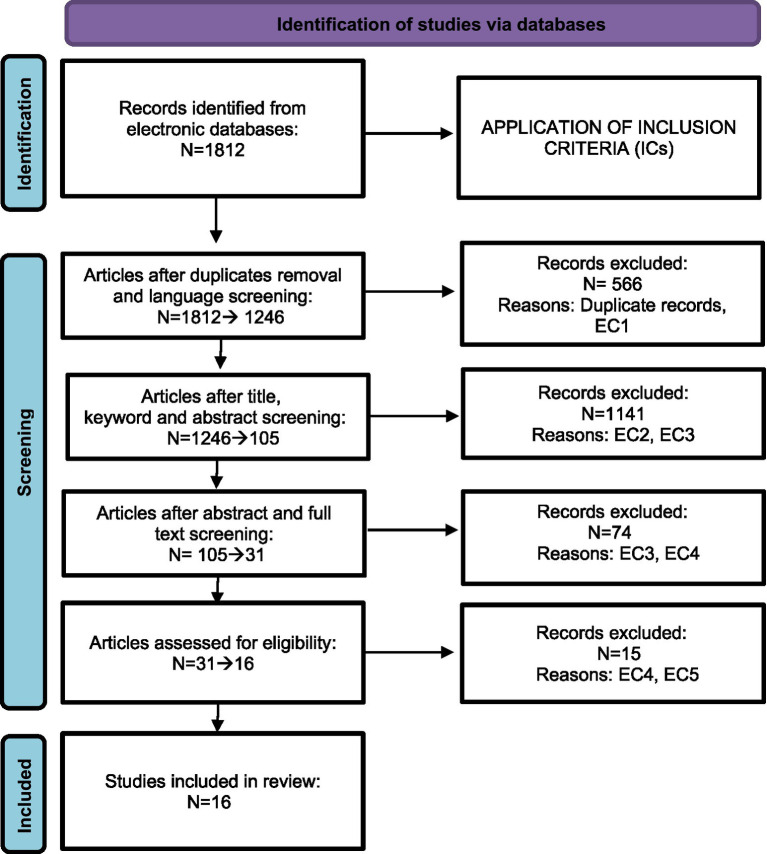
Review process based on PRISMA framework.

Figure 1 illustrates the review process based on the PRISMA framework.

### 2.2 Articles categorization scheme

A full-text analysis of all retained documents was conducted using content analysis. Given the absence of a universally accepted taxonomy of nudges, we developed a categorization scheme to differentiate the types of interventions identified in the reviewed studies. The 16 studies were grouped into five main categories based on definitions and intervention characteristics reported in the respective articles:

- Informational nudges, such as normative messages, framing, labeling, and feedback;
- Reminders and notifications, including emails, letters, and periodic alerts;
- Financial incentives, including monetary rewards and bonuses;
- Non-financial incentives, encompassing symbolic rewards, status recognition, and gamification elements;
- Positioning and default nudges, involving physical or digital modifications to the choice environment.

These categories were developed considering prior conceptualizations of nudging found in the literature (Amiri et al., 2024; Möllenkamp et al., 2019; Ouvrard and Stenger, 2020; Das and Dumortier, 2024). To create these categories, we adopted a Grounded Theory approach (Glaser and Strauss, 1967), as employed by Schwarzinger et al. (2024) in their review of sustainable lifestyle interventions. Grounded Theory, originally proposed by Glaser and Strauss (1967), is widely utilized in qualitative content analysis and well-suited for systematically organizing literature where minimal prior knowledge or predefined hypotheses exist. This bottom-up method facilitates theory development by iteratively coding and grouping raw data—in this case, the corpus of workplace nudging literature—into meaningful conceptual units. The approach follows a recursive logic, enabling agile refinement of coding strategies as new insights emerge. As novel features are identified, they may be incorporated into and expand the evolving conceptual framework. Following Corbin and Strauss (2014), the ideal outcome is a set of well-developed, systematically integrated categories that provide a coherent framework for interpreting the phenomena under study. Grounded Theory is particularly effective for exploring conceptual distinctions across studies, enabling nuanced comparisons of topics, themes, and relationships.

We employed a modification of the original Grounded Theory review approach proposed by Wolfswinkel et al. (2013), applying its five-phase structure. Specifically, during the “Analyze” phase, we employed Grounded Theory’s iterative, inductive logic. In the “Open coding” step, granular preliminary codes were assigned to each publication’s interpretation and application of nudging. Through “Axial coding,” we identified clusters of studies with similar thematic codes and organized them accordingly. Finally, during the final “Selective coding,” we combined the data into an overarching scheme that could not be simplified any further without sacrificing vital information. In the “Present” phase, we synthesized findings to answer the guiding research question: What types of nudges are considered in the retained empirical studies?

It should be noted that no formal quality assessment of included publications was conducted. As a scoping review, the goal was to explore the breadth of existing literature, map key concepts, and identify research gaps, rather than evaluate methodological rigor (Grant and Booth, 2009).

## 3 Results

As shown in Figure 1, we identified a total of 1,812 articles based on the search string. After removing duplicates and applying the inclusion and exclusion criteria, 16 publications were retained for the final analysis. Hereafter, the results regarding our four RQs will be presented. First, we will describe the selected articles in terms of year of publication, geographic area, work sectors, sample size and target behavior, then we will consider the theoretical frameworks referenced in the selected studies, the types of interventions defined as nudges, and their effectiveness in the workplace. In Appendix A, the whole list of the selected articles is provided.

### 3.1 Publication year

Our investigation revealed a substantial increase in research activity on workplace nudges over the past six years. Notably, only one study was identified before 2019 (Kühfuss et al., 2016). In 2019, three relevant studies were published (Peth and Mußhoff, 2019; Byerly et al., 2019; Kristal and Whillans, 2019), followed by two in 2020 (Casado-Mansilla et al., 2020; Kaljonen et al., 2020), four in 2021 (Earnhart and Ferraro, 2021; Dhanorkar and Siemsen, 2021; Klege et al., 2021; Charlier et al., 2021), and six more in 2023, up to May 2025 (Decrinis et al., 2023; Venema and Jensen, 2023; Yang et al., 2025; Hassan et al., 2025; Raineau et al., 2025; Pandey et al., 2025). No articles on this topic were published in 2022.

### 3.2 Geographic area, work sectors, sample sizes, and target behaviors

Most studies were conducted within the European Union and the UK (nine studies), followed by the USA (four studies), and then Canada, South Africa and China (one study each). Regarding the organizational context, three articles focused on activities related to the primary sector including viticulture, cereal farming and forestry (Peth and Mußhoff, 2019; Byerly et al., 2019; Kühfuss et al., 2016), two on the secondary industry, specifically industrial facilities for energy efficiency and automotive (Decrinis et al., 2023; Dhanorkar and Siemsen, 2021), and eight on the tertiary sector, namely catering, waste management, healthcare, transport, utilities and public services (Kaljonen et al., 2020; Earnhart and Ferraro, 2021; Klege et al., 2021; Charlier et al., 2021; Venema and Jensen, 2023; Kristal and Whillans, 2019; Casado-Mansilla et al., 2020).

Notable differences in the sample sizes were detected, ranging from 170 (Kaljonen et al., 2020; Decrinis et al., 2023), to 68,915 workers (Kristal and Whillans, 2019) involved. The levels of analysis included individual or group behaviors, and organizational performances. Three studies featured a mixed sample, including a portion of agricultural science students (Peth and Mußhoff, 2019), other students (Pandey et al., 2025) and hospital visitors and patients (Venema and Jensen, 2023), besides workers. In three studies (Dhanorkar and Siemsen, 2021; Earnhart and Ferraro, 2021; Charlier et al., 2021), the number of participants was not specified, focusing on the entire organization.

The selected articles examined different pro-environmental behaviors in various domains, namely, the adoption of sustainable farming, reduction of pesticides/fertilizers and regulatory emissions compliance (Peth and Mußhoff, 2019; Kühfuss et al., 2016; Earnhart and Ferraro, 2021; Raineau et al., 2025), food choices and waste reduction (Kaljonen et al., 2020; Pandey et al., 2025; Venema and Jensen, 2023), mobility and vehicle choice (Kristal and Whillans, 2019; Decrinis et al., 2023), energy consumption and building efficiency (Casado-Mansilla et al., 2020; Dhanorkar and Siemsen, 2021; Hassan et al., 2025; Yang et al., 2025; Klege et al., 2021; Charlier et al., 2021) and forest habitat conservation (Byerly et al., 2019).

### 3.3 Key nudging references

The analysis of theoretical references in the reviewed literature reveals a stratified conceptual structure, where classical sources on nudging coexist alongside various psychological and behavioral frameworks, resulting in a degree of theoretical ambiguity. Ten out of sixteen studies (Kühfuss et al., 2016; Kristal and Whillans, 2019; Kaljonen et al., 2020; Casado-Mansilla et al., 2020; Earnhart and Ferraro, 2021; Charlier et al., 2021; Dhanorkar and Siemsen, 2021; Venema and Jensen, 2023; Decrinis et al., 2023; Raineau et al., 2025) included in the review cited Thaler and Sunstein (2008), the seminal text that established the theory of nudging and popularized the notion of choice architecture. Additional contributions such as Schubert (2017) and Sunstein (2014) were also cited to reinforce this theoretical foundation by extending the framework to normative and ethical domains. The inclusion of these sources suggests that, at least in principle, many interventions are grounded in an understanding of the cognitive biases and decision-making heuristics that nudges aim to leverage. However, references to these foundational works were often descriptive rather than operational. Only one study (Decrinis et al., 2023) explicitly articulated which specific cognitive mechanism—such as status quo bias, salience, or loss aversion—a given nudge is intended to activate. This lack of specification raises concerns about the replicability and theoretical clarity of the interventions.

Seven studies also draw upon pre-nudge psychological theories that form the deeper conceptual basis of behavioral interventions. One major thread is represented by the literature on social influence, notably the work of Cialdini (1993), Schultz et al. (2007), and Allcott (2011). Although Cialdini does not use the term nudge, his six principles of persuasion—such as reciprocity, authority, and social proof—have been widely incorporated into nudges. Schultz and Allcott provide empirical support for norm-based feedback, demonstrating that individuals’ behavior can be influenced by information about the actions of others. This stream of literature is echoed in the work of Steg and colleagues (Lindenberg and Steg, 2007; Abrahamse and Steg, 2013), who also highlight the role of social norms and social modeling in shaping pro-environmental behavior.

Four out of sixteen studies referred to classical behavioral economics, particularly the work of Kahneman (2011) and Kahneman et al. (1991). These contributions identify cognitive heuristics and biases—such as loss aversion and framing effects—that the nudges used in the respective articles aimed to address. Similarly, Earnhart and Ferraro (2021), referenced Festinger (1954) and his theory of social comparison processes suggesting that some nudges are designed to make salient how individuals’ behavior compares with that of others, thereby motivating them to align their actions with more sustainable norms. Taken together, these references show that many authors draw on well-established psychological principles that underlie behavioral influence. However, it is not always clear how these references are practically translated into nudges (compared to prompts, for instance) neither these theoretical connections are accompanied by specific measurements that would allow researchers to test whether the specific psychological mechanisms have actually been activated.

An emerging and conceptually distinct strand of literature is visible in interventions mediated by digital technologies. In these studies, the theoretical grounding is not behavioral economics, but rather the field of persuasive technology, particularly as articulated by Fogg (2002). For example, Casado-Mansilla et al. (2020) develop nudges embedded in apps, dashboards, or ICT systems based on models such as the Persuasive Systems Design framework and the Fogg Behavior Model, rather than traditional cognitive biases. This shift in theoretical orientation leads to a different logic of design: ICT-based interventions prioritize interactivity, timely feedback, and message personalization, often without explicit reference to choice architecture or behavioral biases. As such, these digital nudging strategies represent a parallel theoretical lineage, which should arguably be treated separately from traditional nudging frameworks when analyzing behavioral interventions.

Finally, it should be noted that two studies (Hassan et al., 2025; Pandey et al., 2025) did not explicitly refer to any theoretical model that directly combines the adopted interventions with the nudging framework.

### 3.4 Types of nudges and their effectiveness

Nudging interventions across the reviewed articles reveal a high degree of diversity, with many studies testing combinations of multiple nudges targeting different sustainability behaviors.

*Informational nudges* and *Reminders and notifications* were employed alone in eight studies (Byerly et al., 2019; Peth and Mußhoff, 2019; Earnhart and Ferraro, 2021; Decrinis et al., 2023; Yang et al., 2025; Raineau et al., 2025; Dhanorkar and Siemsen, 2021) whereas *Positioning and default nudges* alone in one study Hassan et al. (2025). These interventions aimed to increase awareness of environmental consequences or promote compliance with norms. However, their effectiveness varied. For instance, Byerly et al. (2019) sent maple syrup producers letters appealing to descriptive or injunctive norms, but neither increased engagement with the program, even though the descriptive-norm message slightly reduced information requests. In contrast, Earnhart and Ferraro (2021) observed modest reductions in pollution levels after sending letters that combined normative comparisons with injunctive messages, particularly in facilities subject to frequent monitoring. Lastly, a particularly relevant study by Hassan et al. (2025) implemented an ICT-based intervention in 24 offices, subtly adjusting indoor temperature to prompt occupants to close windows when outdoor conditions were unsuitable. This effectively reduced heating demand and minimized inappropriate window openings without compromising perceived comfort. However, windows tended to remain closed even when external air would have been beneficial, highlighting the need for the system to encourage timely reopening.

Some of the studies using *Informational nudges* showed stronger effects when multiple messages were combined. Yang et al. (2025), for example, found that sending WeChat messages blending descriptive and injunctive norms reduced excessive air-conditioning use by encouraging alternatives like lighter clothing and personal cooling devices, whereas single-norm messages had no impact. Similarly, Decrinis et al. (2023) tested email appeals to Porsche employees (emotional, regulatory, or gain-framed) to promote low-emission vehicle selection. All messages were effective if the vehicle order was placed promptly, but only the gain frame had a lasting effect. Interestingly, when the same messages were shown at the point of purchase, their influence disappeared. In the agricultural sector, Raineau et al. (2025) used social-comparison letters to reduce pesticide use: one version reported the cooperative’s mean Treatment Frequency Index (TFI), while another included a histogram of the full peer distribution. After six months, only the average-info version led to behavioral changes, however, these effects faded over time due to extreme weather that reset spraying practices. Peth and Mußhoff (2019) found that emotive posters highlighting nitrate pollution risks improved compliance with buffer-zone rules, while adding a social-norm message reduced the number of violations but increased the severity of the remaining infractions. Dhanorkar and Siemsen (2021), focusing on industrial energy efficiency, found that weekly reminder emails significantly raised the implementation rate of energy-efficiency measures in manufacturing sites. Charlier et al. (2021) showed that combining informational emails with on-site visual prompts (e.g., stickers on devices) reduced electricity use significantly, whereas each component alone was ineffective.

Six studies tested bundled nudges by combining *Informational nudges* and *Reminders and Notifications* with other types of nudges. In three cases, these included *Financial* and *Non-financial incentives* (Kühfuss et al., 2016; Kristal and Whillans, 2019; Klege et al., 2021), in three cases *Positioning and default nudges* (Kaljonen et al., 2020; Venema and Jensen, 2023; Pandey et al., 2025). In only one study, *Informational nudges* were implemented together with *Reminders and Notifications* (Casado-Mansilla et al., 2020).

Kühfuss et al. (2016), for instance, introduced a collective bonus conditional on local participation in herbicide reduction, blending monetary reward with a social pressure component. Kristal and Whillans (2019) tested five interventions including emails with peer testimonials and loss framing, free bus passes, and personalized travel plans, yet observed only minor increases in car-pool registrations and no actual change in commuting behavior. Klege et al. (2021) implemented weekly league-table emails and assigned a rotating “Energy Advocate” in a government building. Emails alone reduced energy by 8%, rising to 13% when responsibility was assigned, although effects diminished over time, suggesting the need for periodic reinforcement to sustain impact.

In restaurant and cafeteria contexts, combining nudges showed more promise. Kaljonen et al. (2020) added climate impact labels to menus and repositioned vegetarian options first in the buffet line. While labels alone had little effect, positioning increased plant-based choices and reduced red-meat consumption. Similarly, Pandey et al. (2025) used default menu options and visual nudges in university cafeterias, raising vegetarian meal uptake and reducing food waste. However, the intervention’s impact declined after the active phase ended. Venema and Jensen (2023) used a “chef’s recommendation” label and eye-level positioning for vegetarian sandwiches. The nudge worked among occasional diners but had no effect on usual staff, indicating that fixed routines may limit the impact of light-touch interventions.

When used together with *Informational nudges, Reminders and notifications* proved effective. Casado-Mansilla et al. (2020) sent weekly emails combining personalized energy use feedback, peer comparisons, and simple tips, leading to a 14% reduction in electricity use. Overall, among the sixteen selected studies, in six cases the application of nudge interventions was effective for all tested nudges (Earnhart and Ferraro, 2021; Klege et al., 2021; Dhanorkar and Siemsen, 2021; Kühfuss et al., 2016; Hassan et al., 2025; Pandey et al., 2025). In one study the application of nudges was ineffective (Byerly et al., 2019), and in the remaining nine studies, the effectiveness of the nudges was mixed, depending on specific factors, such as the characteristics of the subjects or the type of nudges employed.

In general, five main methods were used to assess the effectiveness of the nudges tested across the various studies: (1) objective measures; (2) assessments conducted in controlled environments; (3) self-report questionnaires; (4) focus groups, and (5) a combination of the above-mentioned instruments.

Five studies employed objective measures (Byerly et al., 2019; Earnhart and Ferraro, 2021; Dhanorkar and Siemsen, 2021; Decrinis et al., 2023; Raineau et al., 2025), such as the return of postcards, sales, administrative records, consumption data or environmental indices. Two studies relied solely on self-reported data (Kühfuss et al., 2016; Yang et al., 2025), for example to measure declared land areas or intentions to adopt certain behaviors, attitudes, satisfaction, intentions and commitment. One study (Peth and Mußhoff, 2019) used assessments in a controlled environment, specifically employing a simulation-based game to quantify participants’ responses to interventions, observing changes in water protection behaviors. The remaining eight studies used mixed-method approaches, combining surveys with objective measures.

Twelve articles investigated medium and short-term effects, measuring the effects of the nudging interventions from four weeks to one year following the intervention. Only four studies included long-term measurements beyond the initial treatment period, extending the observations from one to four years after the intervention (Kaljonen et al., 2020; Raineau et al., 2025; Casado-Mansilla et al., 2020; Dhanorkar and Siemsen, 2021).

## 4 Discussion

In recent years, there has been a marked increase in studies examining the application of nudging strategies to promote sustainable behaviors in workplace settings. Notably, more than half of the sixteen included articles were published between 2020 and 2023, reflecting a growing interest from both academic and institutional spheres. The increasing fascination with nudges can be ascribed to their cost-effectiveness and ability to uphold individual choices, paired with the wider demand for effective and inexpensive tools to address escalating social issues (Mehta and Paterick, 2020). However, the research appears still in its infancy, with 16 studies retained out of more than 1,000 found. Most nudging studies addressing organizations have focused on clients, consumers, and service users (Schneider et al., 2020; Gonçalves et al., 2021), somewhat neglecting workers. Recognizing that consumers are not the only contributors to a company’s environmental impact is essential. Businesses and other organizations also influence the environment through their activities, emphasizing the importance of extending attention to workers (Earnhart and Ferraro, 2021).

The interventions primarily focus on reducing energy consumption, promoting responsible resource use, and encouraging more sustainable food choices. Regarding the observed outcomes, six studies report fully positive results, eight indicate partial or heterogeneous effects, and one finds no significant impact. Based on these aggregate findings, it is possible to explore in greater detail the specific characteristics of the studied contexts and the implemented strategies.

A first aspect regards the characteristics of the available studies on nudging to promote workers’ pro-environmental behaviors at work (RQ1). The majority of the studies are set within European or North American organizations operating in the tertiary sector (Evers et al., 2018; Reisch and Sunstein, 2016), with only a few exceptions in manufacturing settings or agriculture. Further research is needed to investigate the implementation of nudges also in other countries, especially those undergoing significant industrialization processes. In such nations, it is crucial for companies to incorporate a high level of environmental awareness from their inception (Gupta, 1995).

The focus on tertiary sector can be justified by the sector’s strong environmental relevance and the high potential for sustainable behavioral change, as these domains involve everyday decisions, such as food choices (Vandenbroele et al., 2019), energy consumption (Jessoe et al., 2020), recycling habits (Saulītis et al., 2024), and transportation modes (Amiri et al., 2024), that can be effectively influenced through subtle behavioral interventions. Moreover, the sector’s direct points of contact with consumers and workers make it a strategic context for applying nudges aimed at promoting more sustainable practices with measurable environmental and social outcomes (Jessoe et al., 2020). In contrast, the number of studies on nudging in the primary and secondary sectors is significantly more limited, although the primary sector is experiencing growing interest. This trend can be attributed to the increasing awareness of the environmental impact of the agricultural sector, particularly regarding emissions, natural resource consumption, and biodiversity loss (Ayeni and Olagoke-Komolafe, 2024).

Nonetheless, the application of nudging interventions in the retained studies spans across all sectors, demonstrating a remarkable degree of methodological flexibility. This cross-sectoral presence reinforces the idea that nudges can be effectively adapted to a wide range of organizational and behavioral contexts (Vandenbroele et al., 2019), regardless of their structural differences or operational dynamics.

A cross-cutting issue emerging from the reviewed studies is the considerable heterogeneity in sample sizes. However, this variability does not correspond to a linear gradient of effectiveness. Large-scale trials, despite their high statistical power, frequently report negligible or null effects, whereas small pilot studies often reveal larger percentage changes, albeit with wide confidence intervals and a higher risk of overestimation. This suggests, according to Szaszi et al. (2022), that sample size alone is not a sufficient explanatory variable, as factors such as the intensity of the nudge, ranging from low-touch mass mailings in large cohorts to personalized or sensor-based interventions in micro-settings, and the level at which outcomes are aggregated (individual versus organizational), play a crucial role in shaping effectiveness. As a result, a trade-off emerges between internal and external validity: small-scale studies offer greater experimental control and insight into psychological mechanisms, but limited generalizability, while large, randomized control trials based on administrative data offer scalable insights for policymakers but often obscure the behavioral processes underpinning the observed effects (Peters et al., 2016). Importantly, three studies (Dhanorkar and Siemsen, 2021; Earnhart and Ferraro, 2021; Charlier et al., 2021), had organizations as the primary unit of analysis, thereby shifting the analytical focus from individual-level behavior to macro-level outcomes. This approach is coherent with policy-relevant indicators (Mazur, 2010), such as environmental performance metrics or compliance rates, which are often defined and measured at the organizational level. However, it substantially complicates direct comparisons with individual-focused studies and limits the interpretability of the psychological mechanisms underlying behavioral change. Indeed, the use of organization-level indicators can mitigate self-report bias but risks masking heterogeneity within the organization, for example, between frontline maintenance staff and upper management (Donaldson and Grant-Vallone, 2002).

From a conceptual standpoint (RQ2), a notable heterogeneity emerges in the retained studies in the use of the term *nudge*. Only half of the studies explicitly refer to the classic definition proposed by Thaler and Sunstein (2008), while the remainder apply the concept implicitly or in a more generic manner, drawing upon well-established theoretical frameworks -i.e., the social norms theory developed by Cialdini (1993), Schultz et al. (2007), and Kahneman (2011) dual-process theory-, to justify the design choices and interpret the observed outcomes.

Thaler and Sunstein’s canonical definition has undoubtedly fostered interdisciplinary dissemination, however, as noted by Hansen (2016) and Grüne-Yanoff and Hertwig (2016), such breadth risks diluting the analytical precision of the concept by conflating it with more intrusive persuasive strategies or veiled economic incentives. Many practical applications now fall outside the boundaries of the original nudge theory, indicating that behavioral tools are often used and designed in ways that diverge from its foundational principles (Tor, 2015).

Approximately half of the reviewed studies report the use of nudges without clearly specifying which bias is being targeted or how the intervention remains “easy and cheap to avoid” (Thaler and Sunstein, 2008; Eyal, 2014). This lack of theoretical transparency and operational clarity is consistent with patterns observed in the broader nudging literature, outside workplace settings (Hansen and Jespersen, 2013), particularly in cases where nudges devolve into forms of sludge (Thaler, 2018), introducing frictions that delay or complicate decisions rather than facilitating them. In light of this previous evidence and the present findings, it is necessary to broaden the traditional theoretical repertoire by integrating emerging approaches such as boosts (Hertwig and Grüne-Yanoff, 2017), dynamic and adaptive defaults (Camilleri et al., 2019), and more structured taxonomies that explicitly connect interventions to their underlying cognitive mechanisms and contextual applications, as highlighted in recent work (Löfgren and Nordblom, 2020). At the same time, the systematic incorporation of ethical analyses (Michaelsen, 2024), addressing issues such as transparency, informed consent, and accountability, is highly desirable. Together, these enhancements would foster a more rigorous operational definition of the nudge concept within contemporary workplace settings. Moreover, this ambiguity hampers the comparability of results across different studies and limits the replicability of interventions. To address these critical issues, the adoption of shared guidelines—such as the PRISMA protocol—is recommended. Such frameworks support a systematic and transparent description of nudging strategies, their theoretical components, and the contextual conditions under which they are implemented.

Moving to the analysis of the types of nudges adopted (Q3), informational nudges and reminders emerged prominently, probably due to their low level of intrusiveness and relatively modest implementation costs. This finding is consistent with previous studies aimed at promoting behavioral change in the fields of health, education (Möllenkamp et al., 2019; Kwan et al., 2020), and consumption, especially in interventions targeting users or consumers (Calzolari and Nardotto, 2011; Binet et al., 2022). Although these nudge categories are the most widely used and can sometimes be effective on their own, they perform optimally when the target behavior involves low costs, and the primary barrier is low cognitive salience (Hotard et al., 2019). However, when behavioral change is impeded by multiple barriers, such as high perceived costs, consolidated attitudes, infrastructural constraints, or opposing social norms (Hauser et al., 2018; Wilson et al., 2015), simply increasing salience is insufficient, and the nudge may fail to yield a significant effect. Moreover, sharing information about others’ involvement can even backfire and reduce interest, particularly when based on inappropriate or insensitive social comparison (Bicchieri and Dimant, 2019; Çat et al., 2024). For these nudges to be effective, it is essential to ensure consistent and clear messaging, provide adequate information dissemination, and incorporate meaningful consequences or incentives to encourage engagement.

Other less-explored nudge categories, such as positioning and default or financial/non-financial incentives, showed considerable promise for application in organizations. Financial and non-financial nudges showed positive effects on shaping social dynamics and inspiring environmentally supportive actions in contexts other than workplaces (Kühfuss et al., 2016). However, they tend to show limited effectiveness when implemented in isolation. Since their strength lies in their ability to enhance the impact of cognitive drivers, particularly when they are aligned with shared values and collective goals (Czap et al., 2018), organizations are proper contexts in which to promote the use of these nudges in combination with others to maximize their impact.

In the retained studies, positioning nudges proved effective only when the environment allowed genuine room for choice, but in highly routinised settings that involve deeply ingrained habits, such as hospitals (Venema and Jensen, 2023) their impact tends to fade (Wachner et al., 2021). This observation underscores the need for greater attention to tailoring nudging strategies based on the contextual constraints, habitual behaviors, and the degree of autonomy available to individuals within the organizations in which they are implemented.

Interestingly, default nudges were observed in only two studies, despite being one of the most well-known and widely used types in other contexts (Lemken, 2021; Paunov et al., 2019). Consistent with the demonstrated effectiveness of default nudges across various contexts (Van Gestel et al., 2021; Van Rookhuijzen et al., 2021), organizations could employ these nudges to incentivize sustainable behaviors, such as automatically enrolling new employees in a green energy program or setting default printer settings to double-sided printing to reduce paper waste.

Finally, with regard to effectiveness and the conditions that influence it (RQ4), our findings suggest that nudges yielded the most favorable outcomes when accompanied by tangible incentives (e.g., rewards or financial savings), when they prompted simple and low-effort actions, and when they were supported by leadership figures or colleagues who reinforced their social legitimacy. Conversely, effectiveness tended to decline when the targeted behavior entailed high perceived costs (e.g., loss of comfort, increased time demand) or when adequate infrastructure was lacking, thereby preventing individuals from acting in alignment with the intended nudge. A growing body of evidence suggests that the effectiveness of nudges cannot be judged along a simple effective/ineffective dichotomy, rather, their impact largely depends on the precise alignment between the behavioral lever, the decision-making, and the contextual characteristics (Zimmermann and Renaud, 2021).

Indeed, although approaches that combine multiple nudges tend to be more robust overall, their use does not always guarantee effectiveness. Adding one or more inputs is only effective if each introduces a distinct behavioral mechanism (Undarwati and Why, 2024); otherwise, it may diminish the overall impact of the intervention resulting in a form of “*overnudging*” (Chapman et al., 2019; Houdek, 2024). Consequently, an ex-ante diagnostic phase is required to systematically map recipients’ cognitive biases, entrenched habits, and the ways each component interacts with the context and prevailing norms, thereby enabling the selection and design of nudges that harness these factors as drivers of pro-environmental actions (Korteling et al., 2023; Undarwati and Why, 2024).

The predominant approach used to assess the effectiveness of nudges in the selected studies was a mixed-methods design, combining objective measures (energy consumption data, recycling rates, or frequency of public transport use) with self-reported data collected through questionnaires and surveys. This approach, as highlighted by several studies (Steele et al., 2024; Jahedi and Méndez, 2014), is considered the most comprehensive, as it allows for a more nuanced understanding of both actual behavioral change and the psychological mechanisms behind it. In contrast, relying solely on a single method, either objective or self-reported, risks overlooking important aspects of the intervention’s impact, leading to partial or potentially biased results.

Furthermore, the lack of long-term evaluations makes it difficult to determine whether the observed changes are enduring or likely to fade once the intervention has ended. The findings reveal a significant gap in data regarding the duration of interventions, highlighting the urgent need to incorporate long-term evaluations and to design longitudinal studies capable of identifying the optimal frequency and modalities of reinforcement (Zimmermann and Renaud, 2021). Only through such an approach will it be possible to develop a more robust theoretical understanding of behavioral change dynamics and to provide practical guidance aimed at achieving truly sustainable and lasting outcomes.

Despite the promising findings, the reviewed studies present several limitations that warrant consideration. First, the geographic concentration in high-income Western countries and the tertiary sectors constrain the generalizability of the results to other cultural, economic, or organizational contexts. In addition, the considerable heterogeneity in methodological designs, intervention types, and outcome measures limits the feasibility of systematic comparisons or meta-analytical synthesis. Another important limitation is the scarcity of long-term follow-up assessments, which makes it difficult to evaluate the persistence of effects over time.

The present review also presents some limitations which have to be acknowledged. First, the decision to include only peer-reviewed and openly accessible resources—while excluding gray literature and subscription-based materials unavailable to the authors’ affiliated institution—may have limited the overall comprehensiveness of our search. Furthermore, while English is generally considered the universal language of science (Morrison et al., 2012), exclusive reliance on English-language studies may not represent all the evidence. However, as demonstrated by Dobrescu et al. (2021) and Morrison et al. (2012) in the medical field, limiting literature reviews to English-language publications seems to exert minimal influence on the effect estimates and overall conclusions derived from such reviews. Finally, although we applied systematic inclusion criteria, the process of article selection and categorization in this review also has inherent limitations, as some subjectivity may arise. Adopting a three-authors independent review of search results can help mitigate this risk (Stoll et al., 2019), but it cannot be eliminated.

## 5 Conclusion

This review examined the use of nudges in organizational settings to promote pro-environmental behaviors among workers. The results indicate that nudging within workplace is a new and evolving field of research, and not all nudge types are equally represented in the current literature. A critical priority is to articulate a precise definition of “nudging,” which remains insufficiently delineated, in order to ensure that interventions align with its conventional conceptualization and to expand the research agenda to encompass emerging studies and innovative approaches. Organizational nudging must also acknowledge that driving behavioral change within organizations involves more than influencing individual choices—it also requires reshaping the social and structural conditions under which those choices occur (Mols et al., 2015). Consequently, behavioral insights should be integrated into the very fabric of organizational design—including processes, roles, and norms. This approach calls for a comprehensive understanding of how work is structured, how information circulates, and how decisions are made, ensuring that these components collectively foster and sustain the targeted behavioral outcomes (Houdek, 2024).

In light of these considerations, future research would benefit from the development of longitudinal studies conducted in non-Western countries, the adoption of shared operational definitions for nudging strategies, and the exploration of integrated interventions that also incorporate structural changes to the environment. These research directions could contribute to building a more robust and applicable framework for designing sustainable work environments and fostering the widespread adoption of ecologically responsible behaviors—both within and beyond workplace settings, thanks to a positive cascade effect.
