# Supplementary material for: More sustainable choices in the workplace: a systematic review of nudge theory applications
Source: Front Psychol. 2025 Aug 20;16:1556796. doi: 10.3389/fpsyg.2025.1556796 (PMC12406869; doi:10.3389/fpsyg.2025.1556796)
Supplement: Supplementary file 2 [file Supplementary_file_1.docx]

**Appendix A.** *List of the selected studies in the data extraction form.*

| **Author(s)** | **Context & Sector** | **Sample** | **Unit of Analysis** | **Key Nudging References** | **Type(s) of Nudge** | **Interventions** | **Effectiveness and Target Behaviors** | **Assessment Methods** |
| --- | --- | --- | --- | --- | --- | --- | --- | --- |
| **Kuhfuss**  **et al.,**  **2016** | Viticulture in Languedoc-Roussillon, southern France, where herbicide use threatens groundwater quality. | 290 wine-growers (about seven thousand hectares). | Individual | Thaler & Sunstein (2008) | Informational nudges + Financial nudges | Conditional collective bonus of one hundred and fifty euros per hectare paid only if at least 50% of the local vineyard area joins; combines descriptive and injunctive social norms with a modest monetary incentive. | • Short-term: willingness to accept falls by roughly one hundred ten to one hundred forty euros per hectare per year and predicted enrolled area rises by about two hundred sixty-five hectares (an increase of four percentage points). • Long-term: effects were not measured. | Online discrete-choice experiment with six choice cards; responses analysed via mixed-logit and two-stage models. |
| **Peth**  **&**  **Mußhoff, 2019** | Eight-period online arable-farming simulation reflecting German crop farms subject to a legally binding three-metre buffer from surface water. | 163 farmers and 144 agricultural-science students. | Individual | Schultz et al. (2007); Schubert (2017) | Informational nudges | Empathy-oriented message with emotive pollution images; Social-comparison message adding that most farmers in the area comply. | • Short-term: empathy nudge reduced non-compliance from about eighteen to 5% among students and from 17% to 9% among farmers. The social-comparison nudge reduced non-compliance from 25% to 14% among students and from 16% to 9% among farmers, but increased the illicitly fertilized area among those who still violated the rule. • Long-term: effects were not measured. | Behavioural log files from an eight-round online farm-management game. |
| **Byerly**  **et al.,**  **2019** | Private “sugarbush” maple-syrup operations in the Northern Forest of Vermont, United States. | 967 Maple Producers | Individual | Cialdini et al. (1991); Abrahamse & Steg (2013); Allcott (2011) | Informational nudges | •Descriptive norm message: “Many of your fellow sugar makers are part of the Bird-Friendly Maple Project.”  • Public-recognition message: “Recognizing the stewardship of sugar makers through the Bird-Friendly Maple Project.” | • Short-term: information-request rate dropped from 18.6% in the control to 12.8 % with the descriptive norm, a decline of six percentage points. The recognition letter yielded sixteen-point-one percent, not significantly different from the control.  • Long-term: effects were not measured. | Postal, three-arm field RCT; postcard returns recorded by researchers. |
| **Kristal**  **&**  **Whillans, 2019** | A large international airport outside a major European city, with free on-site parking yet extensive bus, rail, and car-pool infrastructure. | 69,145 workers (Study 1: 54,887; Study 2: 871; Study 3a: 7,560; Study 3b: 4,732; Study 4: 1.095) | Individual | Thaler & Sunstein (2008); Sunstein (2014); Allcott (2011); Kahneman & Tversky (1979) | Informational nudges + Reminders and Notifications + Financial incentives | Behaviourally informed letters reducing friction and featuring peer testimonials; targeted emails with personalised car-pool matches plus opportunity-cost reminders; a small incentive in the form of a free seven-day bus pass; a loss-framed reminder letter; personalised multimodal travel plans with discounts. | • Short-term: Across all trials, effect sizes ranged from minus zero point zero one to plus zero point zero five; four of five studies were statistically equivalent to no effect;  • Long-term: persistence beyond 2 months not assessed | Employer records of car-pool and transit use, email click-through metrics, and a brief post-intervention survey. |
| **Kaljonen**  **et al.,**  **2020** | Workplace canteen at the Finnish Environment Institute in Helsinki; about 250 lunches served per day by a commercial caterer. | 170 employees (pre-intervention survey), 24 employees (focus group) | Individual / meal sales | Thaler & Sunstein (2008); Kahneman (2011) | Informational nudges + Positioning nudges | Informational climate label on menus and buffet; choice-architecture change that placed more varied and tastier vegetarian dishes first in the serving line. | • Short-term and Long-term: From 2014 to 2017, vegetable purchases rose about 10% and pulses about 33%; a clear drop in meat occurred only in 2017. Average meal emissions stayed near 1.8 kg CO₂-e because added dairy offset lower meat. | Canteen sales-and-purchase database merged with FoodWeb LCA outputs, complemented by pre/post surveys and focus groups. |
| **Casado-Mansilla et al., 2020** | Seven office-type buildings (universities, municipal offices, innovation centres) in Spain, the United Kingdom, Greece, and Austria, within the EU H2020 GreenSoul project. | About 350 workers | Individual | Thaler & Sunstein (2008); Cialdini (1993); Fogg (2002) | Informational nudges + Reminders and Notifications | Self-monitoring via an interactive desktop coaster showing real-time consumption; conditioning through a gamified mobile app awarding points; cause-and-effect messaging with posters linking actions to energy/CO₂ impact. | • Short-term and Long-term: Action-stage participation rose by 8.5 percentage points overall; best scenario modelling suggests up to 25 % energy saving potential. | Thirty-six-item online pre-survey, streamlined post-survey, and GreenSoul IoT meters on personal and shared devices. |
| **Earnhart & Ferraro, 2021** | Municipal wastewater-treatment facilities are regulated under the U.S. Clean Water Act in Kansas. | 328 active municipal plants | Company sites | Festinger (1954); Schultz et al. (2018); Thaler & Sunstein (2008) | Informational nudges | One certified-mail peer-comparison letter that displayed each facility’s discharge ratio and percentile relative to all Kansas plants, conveying both descriptive and injunctive social norms. | • Short-term and Long-term: Discharge ratio fell by about 8% overall; three of six quarters showed significant drops, with no consistent trend. Monitoring ended at eighteen months. | Mandatory Discharge Monitoring Reports matched to permit limits. |
| **Klege et al., 2021** | Twenty-four-storey provincial government office building in Cape Town, South Africa, equipped with floor-level smart meters recording half-hourly electricity use. | About 1000 workers (twenty-one floors) | Individual floor for energy data; individual employee perspectives in interviews. | Abrahamse et al. (2005); Allcott & Kessler (2019); Allcott & Mullainathan (2010); Allcott & Rogers (2014); Schultz et al. (2007) | Reminders and Notifications + No-financial nudges | Action-oriented reminder emails with simple conservation tips, weekly league-table emails comparing floors’ energy use, and assignment of a rotating floor “energy advocate”. | • Short-term and Long-term: Over the five-month study, Treatment I cut average use by about 8% and Treatment II by about 13%, though effects declined from approximately 21% and 15% in the first month to those average levels by month five. | 30-minute floor-level smart-meter logs, automated email-delivery records, and post-intervention qualitative interviews with occupants. |
| **Charlier et al., 2021** | Forty-seven office sites of the same French company, each with a building-management system that records daily electricity and heating demand. | 47 company sites | Company sites for energy data; individual employees for survey attitudes. | Thaler & Sunstein (2008); Allcott (2011); Allcott & Rogers (2014); Cialdini et al. (1990) | Informational nudges | Moral appeal e-mails describing global-warming harms; Social-comparison reports ranking each site’s consumption against peers; Visual prompts in the form of good-practice stickers on thermostats, switches, and printers.  Two combined treatments paired stickers with either the moral appeal or the social comparison. | • Short term: Weeks five to eight: combined treatments lowered daily consumption by roughly seven to eight kilowatt-hours per site relative to the control.  Weeks nine to twelve: social-comparison plus stickers cut use by about forty-five kilowatt-hours per site per day.  • Long-term: effects beyond the twelve-week window were not measured. | Building-management-system logs of daily electricity and heating use, plus pre- and post-employee surveys on perceived behavioural change. |
| **Dhanorkar &**  **Siemsen, 2021** | Manufacturing facilities in the United States that receive technical-assistance audits from the Minnesota Technical Assistance Program (MTAP); after each audit, plants decide whether and when to carry out the recommended efficiency actions. | 134 manufacturing plants | Company sites | Thaler & Sunstein (2008); Allcott & Rogers (2014); Allcott & Mullainathan (2010) | Reminders and Notifications | Reminders (e-mails or phone calls) are sent at varying frequencies. | • Short-Term and Long-Term: - Archival analysis: shifting from quarterly to monthly reminders raised the implementation hazard about five-fold and lifted completion probability from roughly 33% to about 40%.  -Field study: weekly reminders sustained higher completion rates throughout 2014-2017 (visual and regression evidence). Durability beyond eighteen months was not observed. | MTAP audit database combined with time-stamped reminder logs, analysed using hazard models and instrumental-variable probit; follow-up difference-in-differences for the policy rollout. |
| **Venema**  **&**  **Jensen, 2023** | Regional hospital café in Denmark that serves both staff and visitors in a real-world setting. | 186 hospital staff, patients, and visitors (+ sales logs covering twenty-four thousand eight hundred sixteen sandwiches) | Individual | Thaler & Sunstein (2008) | Informational nudges + Positioning nudges | A bundled salience treatment that included a chef-recommendation sticker on vegetarian sandwich bags, eye-level display with a sustainability sign, placement of vegetarian items first in the visual line, and more colourful packaging. | • Short-term: During the fourteen-day intervention, the vegetarian share rose from about sixteen to 25% overall, from roughly 14 to 28% for visitors, and about nineteen to 22% for staff.  • Long-term: No data were collected beyond the two weeks. | Cash-register records flagged as staff or visitor (fixed vegetarian-to-meat ratio), plus exit survey on sticker notice, café-visit frequency, and a five-item meat-habit scale. |
| **Decrinis**  **et al.,**  **2023** | Porsche headquarters in Germany; employees choose a new company or lease a car each year through an online Employee Car Configurator. | 170 employees | Individual | Thaler & Sunstein (2021); Kahneman (2012); Kahneman et al. (1991); Lindenberg & Steg (2007); Cialdini et al. (1991) | Informational nudges | Framed e-mails sent eight months before the car change and matching pop-up messages, using one of three frames: Emotional – “The heart electric, the soul Porsche” + sports-car imagery.  Normative – “Be an ambassador for a sustainable Porsche future.”  Gain – “Swap petrol for electricity and cut monthly running costs by €100." And pop-up with the same three frames | • Short-term: Immediate EV share reached 82% under the gain frame, 66% under the normative frame, and 64% under the emotional frame, compared with 53% in control. The gain frame raised the odds of choosing an EV by about three-point-seven times and remained positive for roughly seven weeks, whereas other frames faded after three to four weeks.  • Long-term: No data beyond the ordering window. | Automatic transaction logs from the Employee Car Configurator were analysed with logistic regression. |
| **Yang et al., 2025** | Online experiment with finance-sector employees in Guangdong Province, China, framed as a WeChat group-chat discussion about raising the office air-conditioning set-point. | 743 employees | Individual | Cialdini & Trost (1998); Cialdini et al. (1990); Schultz et al. (2015) | Informational nudges | Three different messages: Descriptive norm: “80 % of Guangdong finance workers already adjust clothing, 60 % use PCDs.”; Injunctive norm: company leader reply endorsing personal cooling; Combined: both messages shown together. | • Short-term: During the immediate post-message survey, the combined norm reduced average clothing-warmth intention by about zero point one six on a five-point scale and raised high-commitment odds two-point-four times versus control. Single norms produced no improvement and reduced commitment. No effects were found for personal cooling devices.  • Long-term: No follow-up was conducted. | Online pre- and post-survey responses; intervention delivered through a mock WeChat screenshot. |
| **Hassan et al., 2025** | Mixed-mode “living-lab” office building in Ottawa, Canada, with twenty-four single-occupant rooms; building alternates between natural and mechanical ventilation. | 24 private offices, each occupied by a single individual, primarily academic or administrative staff. | Individual | N/P | Default nudges | The building-management system raises the set-point in summer or lowers it in winter when a window is left open during mechanical conditioning, making the room briefly less comfortable and signalling the occupant to close the window. Normal settings resume once the window is shut. | • Short-Term and Long-Term: During the post-intervention period, heating energy fell by roughly 35% and windows remained closed after the first nudge event, indicating a carry-over effect. Effects were tracked for one full heating-and-cooling season. | Building-automation logs of window status, indoor/outdoor climate, and HVAC energy use, plus a post-season online comfort survey. |
| **Raineau et al., 2025** | Bordeaux wine-grower cooperative in France facing pressure to cut pesticide applications. | 247 growers | Individual | Thaler & Sunstein (2008); Schultz et al. (2007); Allcott (2011); Allcott et al. (2019); Allcott & Rogers (2014); Kahneman et al. (1991) | Informational nudges | Two certified-mail letters: 1) Average letter: own Index with cooperative mean.  2)Full-distribution letter: same information plus a histogram of the entire distribution. | • Short term, crop year twenty sixteen:  -The whole sample saw about a five per cent reduction versus the control under the average letter, not significant overall.  - Heavy users, defined as at least two points above the mean at baseline, cut their Index roughly 15% more than controls, marginally significant.  - Growers in the strongest reduction quartile were 16 percentage points more likely to be in the average-letter group than in control, but not in the full-distribution group.  • Long term: Differences vanished by twenty nineteen, probably because extreme weather in twenty seventeen and twenty eighteen and learning decay erased early gains | Annual mandatory spray logs supplied by the cooperative and letter-delivery records. |
| **Pandey et al., 2025** | Three education-sector canteens (universities/vocational colleges) in Denmark; pre–post quasi-experiment. | 508 workers/students (thousand eight hundred seventy meal purchases) | Individual | N/P | Informational nudges + Positioning and Default nudges | A bundle of low-cost visual primes (table-top cues, floor stickers, signage) strategically positioned between the meat option and the plant-based “target” meal | • Short term: During the intervention fortnight, plant-based purchases rose 38% and meat choices fell 19%; total plate waste dropped by about sixty kilogrammes, a per-plate decrease of roughly 6.5 %.  • Long term: No data were collected beyond the intervention period | Point-of-sale transaction logs, weighed waste buckets, and pre/post online surveys; per-plate carbon footprint estimated via life-cycle assessment. |
|  |  |  |  |  |  |  |  |  |
